# Aesthetic experiences across visual perception and mental imagery: Behaviorally indistinguishable, neurally distinct

**DOI:** 10.1016/j.isci.2025.112588

**Published:** 2025-05-05

**Authors:** Maximilian Kathofer, Claus Lamm, Helmut Leder, Julia Sophia Crone

**Affiliations:** 1 Vienna Cognitive Science Hub, University of Vienna, Vienna, Austria; 2 Social, Cognitive, and Affective Neuroscience Unit, Department of Cognition, Emotion, and Methods in Psychology, Faculty of Psychology, University of Vienna, Vienna, Austria; 3 EVA-Labs, Department of Cognition, Emotion, and Methods in Psychology, Faculty of Psychology, University of Vienna, Vienna, Austria

**Keywords:** Behavioral neuroscience, Cognitive neuroscience

## Abstract

Studies suggest that vivid mental imagery can blur the boundaries between reality and imagination in simple detection tasks by eliciting similar neural patterns. The question arises as to whether aesthetic experiences can similarly emerge through imagery or whether these complex experiences necessitate direct input into the sensory system. Alternating between visually perceiving and reimagining encountered stimuli, 34 participants rated faces and artworks across aesthetic dimensions. While slightly less potent, imagery is equally sufficient in evoking aesthetic experiences, and for highly vivid imaginations, evoked experiences even become behaviorally indistinguishable across conditions. Yet, representational similarity analysis reveals distinct neural patterns across conditions as the brain mainly encodes stimulus modality, even in areas canonically associated with aesthetic processing. Thus, although evoked experiences might be behaviorally identical across modalities during highly vivid trials, neural patterns differ substantially due to differences in modality, as evoked experiences are only marginally encoded.

## Highlights

• Visual imagery is sufficient to elicit profound aesthetic experiences
• Highly vivid imagery elicits aesthetic experiences indistinguishable from visual ones
• Despite behavioral similarities, neural patterns differ across conditions
• Even within the reward system, neural patterns mainly encode differences in modality

## Graphical abstract

**Figure:**
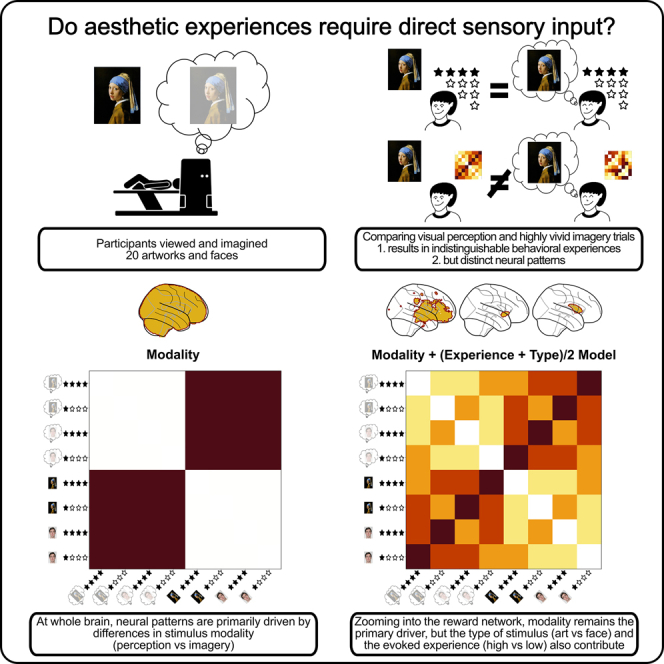

## Introduction

The aesthetic experience is a fundamental human trait that shows considerable cultural variability.^1^ Deeply rooted within our sense of pleasure,^2^ neuroaesthetics employs the aesthetic experience as an umbrella-term for a wide range of cognitive concepts, emotional states, and their brain responses, encompassing nuanced facets, such as beauty, the sublime,^3^ and the state of being moved.^4^ Yet, despite its crucial role in shaping human experiences, finding a universal definition of the aesthetic experience has continuously challenged philosophers, psychologists, and neuroscientists alike.^5^ At the core of this debate is whether aesthetic experiences are a quality of the perceived object itself or whether they emerge from an internal state reflecting the complex interplay between the inducing sensory properties of an object and ongoing internal cognitive processes.^5^^,^^6^^,^^7^^,^^8^^,^^9^ While there is prevailing consensus that aesthetic experiences stem from first-hand encounters, it is also well established that aesthetic experiences are more than just the mere product of the directly perceived stimulus properties, and thus, are affected by factors beyond characteristics of the external sensory stimulation. For instance, peak aesthetic moments, experiences of awe or chills, are profoundly influenced by cognitive factors such as meaningfulness^10^^,^^11^^,^^12^ and ongoing thought processes.^2^ More direct evidence against the notion that the aesthetic experience is merely the result of the properties of an external stimulation comes from a study investigating the complexity of the brain network response to music.^13^ The more rewarding the music was, that is, the higher the aesthetic experience, the less did the complexity of brain network dynamics match the complexity of the music. This strongly indicates that the processes underlying the emergence of an aesthetic experience involve more than just the original stimulation properties. In consequence, the question arises whether the emergence of an aesthetic experience even necessitates the immediate external perception of an object, such as viewing an artwork, or if direct physical input to the sensory system is merely one of several effective means to evoke such an experience, with internally generated representations being equally sufficient. While it may seem intuitive, given the essential role attributed to subjectivity and cognitive processes in aesthetic experiences, the significance of the external stimulus perception for aesthetic processing has surprisingly never been evaluated in empirical research. Answering the question whether the external perception is necessary and how much it contributes to an experience, however, has broader implications for understanding how experiences emerge in general from the interaction between the external world and the internal processes of each individual.

If indeed external stimulation merely acts as a trigger initiating internal, subjective processes which are responsible for the aesthetic experience or, for that matter, any experience at all, then mental imagery (the ability to voluntarily represent objects in the mind in the absence of immediate external stimulation of the sensory system) should also lead to an aesthetic experience of similar quality. Neuroimaging studies indicate that visual mental imagery largely recruits brain regions commonly engaged in visual perception.^14^ More specifically, there is a significant overlap of neural activation particularly within the ventral visual stream between visual mental imagery and visual perception, with more pronounced similarity of activation patterns for higher visual areas, yet, nuanced differences for earlier visual regions.^15^ This is unsurprising as activation in these lower-level areas is significantly affected by the level of detail of the imagined object as well as the vividness of the mental image itself.^16^^,^^17^^,^^18^^,^^19^ Vividness is defined as the degree of clarity of the mental image^20^ and individuals significantly differ in their ability to represent objects in their mind with some lacking the ability to perform visual mental imagery completely.^21^^,^^22^ Depending on the imagined content, mental imagery can evoke both positive and negative affective responses comparable to those triggered by visual perception,^23^^,^^24^^,^^25^ by similarly recruiting key emotional and reward-related regions, such as the amygdala and the nucleus accumbens.^26^^,^^27^^,^^28^ Involuntary imagery even contributes to psychopathological symptoms, most notably in post-traumatic stress disorder, in which intrusive flashbacks elicit intense emotional distress and serve as a hallmark of the disorder itself.^29^ Additionally, adverse imagery contributes to various other clinical conditions,^30^^,^^31^ driving cravings,^32^^,^^33^ and maladaptive behavior.^34^ Thus, due to its profound impact on affective processing, clinicians leverage techniques such as imagery rescripting^35^ and neurofeedback^36^ to fundamentally reshape emotional responses to distressing mental images and modulate subjective emotional processing long-term.^37^^,^^38^ Taken together, external sensory stimulation and mental imagery are deeply intertwined, engaging overlapping brain regions and often producing remarkably similar phenomenological experiences.

In light of these qualitative and neural similarities, a long-standing question has centered on the precise mechanism enabling the brain to reliably distinguish visual perception from mental imagery. Recently, differences in neural signal strength emerged as a potential mechanism facilitating this distinction.^39^ These findings suggest that if the strength of neural representations is high enough, vivid mental images might even be perceived as real. Acknowledging the potency of mental imagery in shaping our perception, it seems highly plausible that mental images themselves should also be able to evoke more complex second-order effects such as aesthetic experiences. Thus, if an aesthetic experience does not necessitate external sensory stimulation and if there was no unique contribution of the external sensory stimulation to shaping the aesthetic experience, the quality of the aesthetic experience itself should not substantially differ whether they are sparked by external stimulation or by mental imagery of the same aesthetic stimulus. In this case, both stimulation modalities (external visual perception and internal visual mental imagery)—if controlled for vividness—should result in phenomenologically very similar subjective aesthetic experiences.

Lastly, since neural activity in the reward network and more precisely the nucleus accumbens and the caudate nucleus are closely associated with aesthetic processing,^40^^,^^41^^,^^42^^,^^43^ activity in these highly specialized areas should primarily reflect the nature of the aesthetic experience itself. In contrast, in areas such as the visual cortex, neural patterns are significantly shaped by modality, even though the neural response to external visual perception and mental imagery becomes more similar as the vividness of the mental image increases.^17^ Thus, if mental imagery can evoke subjective experiences phenomenologically similar to those triggered by external stimulation, we expect that these similarities would be encoded in the neural activity of these highly specialized areas rather than the differences between modalities in which the stimulus was originally perceived.

The present study aimed to provide empirical evidence for the question of whether the emergence of an aesthetic experience truly necessitates external sensory stimulation or whether it can equally arise in its absence, solely triggered through internally generated images. First, we created an aesthetic task utilizing a diverse range of both cultural and biological pre-rated stimuli—artworks and faces—presented across visual perception and mental imagery conditions, to examine whether aesthetic experiences can similarly arise in response to mental imagery (hypothesis 1 (H1)). Crucially, to capture a broad range of aesthetic responses, participants rated evoked experiences across three dimensions; pleasure (which reflects the extent to which the experience was liked), beauty (a measure closely linked to pleasure but reliant on cognitive assessment of the stimulus and its properties^2^), and being moved (a strong affective response that can even trigger physiological reactions, such as chills^44^). In line with our hypothesis, we indeed find that imagery can elicit the full range of aesthetic experiences similarly to visual perception, albeit to a lesser extent. Next, we investigated whether naturally occurring trial-by-trial variability of an individual’s proficiency to vividly imagine a stimulus^17^ might influence evoked responses (H2). We not only show that aesthetic experiences become more similar across modalities dependent on the clarity of the mental image but also that aesthetic experiences become virtually indistinguishable across modalities for highly vivid trials. Lastly, given the suspected similarity of aesthetic experiences across modalities, we reasoned that the neural signal underlying these experiences should be similar as well (H3). More specifically, we expected that the dominant stimulus feature encoded in the neural signal, i.e., modality, type, or experience, would vary depending on the level of resolution, that is, whole-brain, reward network, or highly specialized regions within the reward network, such as the nucleus accumbens and caudate nucleus. Whereas global neural patterns were expected to differ significantly across modalities—despite similarities in the evoked experience—due to the inclusion of primary visual areas, the ventral and dorsal pathways, neural patterns in highly specialized regions were predicted to be highly similar, reflecting shared behavioral responses despite differences in stimulation modality. Testing several theory-driven models using representational similarity analysis (RSA), we found that at the global level, neural patterns exclusively encoded modality differences. However, as we zoomed into the reward network and its constituents, the neural signal increasingly reflected evoked experiences and stimulus type. Nevertheless, modality differences remained the dominant factor, contributing twice as much to the neural signal. Ultimately, this shows that the neural signal still largely differs across conditions, and despite a highly similar behavioral experience, does not need to be identical.

## Results

### Highly vivid imagery elicits indistinguishable aesthetic experiences from visual perception

**Figure 1:**
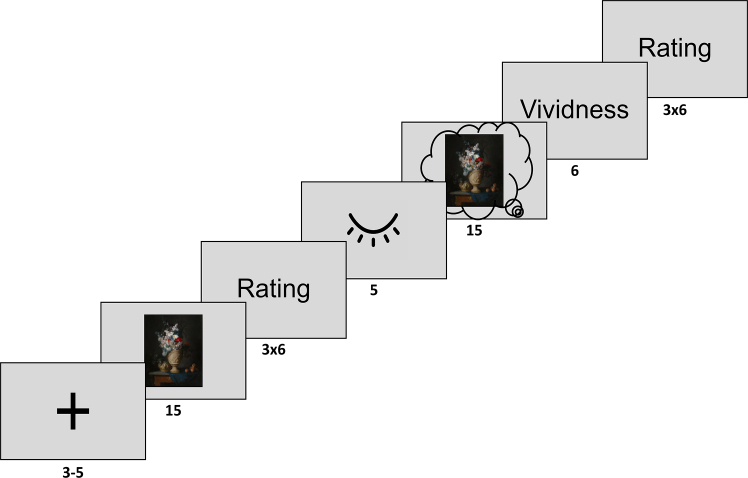
Aesthetic task Participants were randomly presented with 40 pictures of representational artworks and faces. First, participants directly viewed the stimulus for 15 s and rated the evoked aesthetic experience across three scales: pleasure, moving, and beauty. Next, participants closed their eyes and imagined the previously encountered stimulus for 15 s. Lastly, the vividness of the mental image was assessed as well as the evoked experience in response to the mental image. Durations are in seconds.

To determine whether external sensory perception is necessary for the emergence of an aesthetic experience (see H1), participants (*N* = 34) performed an aesthetic task in which they rated their subjective aesthetic experience in response to images of faces and artworks across a visual perception and a mental imagery condition (see Figure 1). To assess whether visual perception is necessary or mental imagery is equally sufficient in eliciting profound aesthetic experiences, we compared the ratings of evoked experiences across different stimulation modalities (visual perception and visual mental imagery) for each stimulus type (faces and artworks). Crucially, to capture a plurality of aesthetic dimensions, participants rated evoked experiences across three key facets: pleasure, which reflects the extent to which a stimulus evokes hedonic experiences; beauty, a measure closely linked to pleasure but reliant on cognitive assessment of the stimulus and its properties^2^; and being moved, a strong affective response that can even trigger physiological reactions, such as chills.^44^

In line with our initial hypothesis that mental imagery is equally capable of eliciting aesthetic experiences, Bayesian ordinal regressions highlight that visual mental imagery elicits aesthetic experiences comparable to those evoked by visual perception, albeit to a lesser extent: moving (*β*_*stand*_ = −0.29 [CI_95_: −0.37–0.21] (see Table S1); beauty (*β*_*stand*_ = −0.28 [CI_95_: −0.37–0.20]) (see Table S2); pleasure (*β*_*stand*_ = −0.22 [CI_95_: −0.31–0.14] [see Table S3]). Suspecting that naturally occurring trial-by-trial fluctuations in participants’ vividness abilities might drive these small differences across stimulation modalities (see H2), we calculated an absolute similarity score between aesthetic responses across modalities. This was done by subtracting each individual rating in the imagery condition from its visual counterpart, squaring it, and then adding them together for each stimulus per subject. This resulted in a vector ranging from 0 (perfect similarity) to 48 (highest observed dissimilarity). Using a Bayesian linear model, we find a clear negative association between the vividness of mental images and the dissimilarity of responses (*β*_*stand*_ = −0.27 [CI_95_: −0.40–0.14] [see Tables S4.1 and S4.2]), indicating that differences in evoked experiences across modalities became larger when mental images were less precisely imagined.

**Figure 2:**
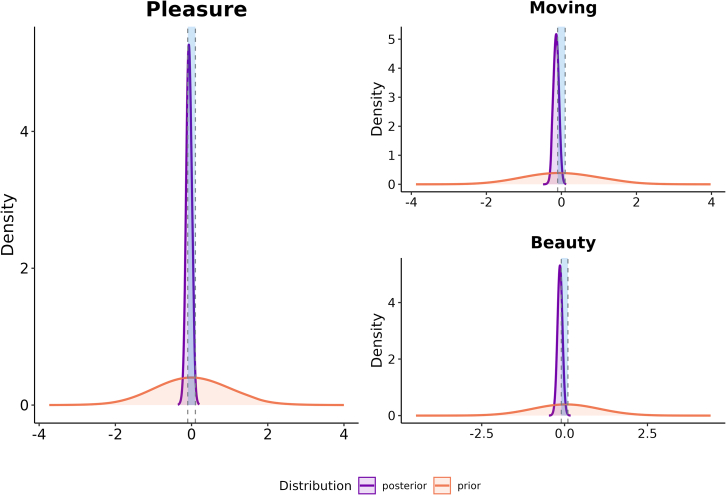
Bayes Factor analysis for parameter estimates of highly vivid trials Each plot shows parameter estimates of the ordinal regression. Plots show prior distributions (green) and posterior distributions of the parameter estimate (red) across all 3 aesthetical facets as well as the range of effect sizes generally considered not meaningful (gray).

Thus, to substantiate the claim that both stimulation modalities—when controlled for vividness—evoke phenomenologically equivalent subjective aesthetic experiences, we conducted a subgroup analysis using only trials with the highest vividness ratings (6 and 7) for each participant. Across all 3 facets, the observed differences across stimulation conditions became exceedingly small, and most importantly, all effect size estimates now included 0 as a probable value for the effect size estimates: moving (*β*_*stand*_ = −0.14 [CI_95_: −0.29 0.01] [see Table S5]); beauty (*β*_*stand*_ = −0.14 [CI_95_: −0.29 0.01] [see Table S6]); pleasure (*β*_*stand*_ = −0.07 [CI_95_: −0.22 0.07] [see Table S7]). Further harnessing the advantages of the Bayesian framework, we then calculated a Bayes Factor for each of these parameters to assess whether their posterior distribution shifted away or toward a region of effect sizes typically deemed as negligible. This region traditionally ranges from −0.1 to 0.1,^45^^,^^46^ as effects of this size are generally deemed insignificant. For all three aesthetic facets, we find moderate to strong evidence^47^ that observed effects fall into this range of negligible effect sizes (*BF*_*01-*moving_ = 4.6; *BF*_*01-beauty*_ = 4.7; *BF*_*01-pleasure*_ = 22.7) (see Figure 2). Given that the detected effect sizes are exceedingly small and would be typically considered as negligible within the field of psychology, we conclude that there is no compelling evidence that visual mental imagery results in meaningfully different aesthetic experiences compared to visual perception during highly vivid trials. Thus, when controlled for vividness, evoked aesthetic experiences across stimulation modalities are largely indistinguishable from each other.

In addition, we used two different types of stimuli to induce an aesthetic experience; biological stimuli, that is, faces and cultural stimuli, that is, artworks. The results clearly indicate that faces elicited weaker experiences than representational artworks across all three facets of aesthetic experience (moving [*β*_*stand*_ = −0.56 [CI_95_: −0.77–0.35]) (see Table S1); beauty (*β*_*stand*_ = −0.43 [CI_95_: −0.71–0.13]) (see Table S2); pleasure (*β*_*stand*_ = −0.82 [CI_95_: −1.06–0.58]) (see Table S3), even though we used a pre-rated set of stimuli for both.

### Neural similarity patterns, even in regions within the reward network, are mainly driven by stimulation modality

**Figure 3:**
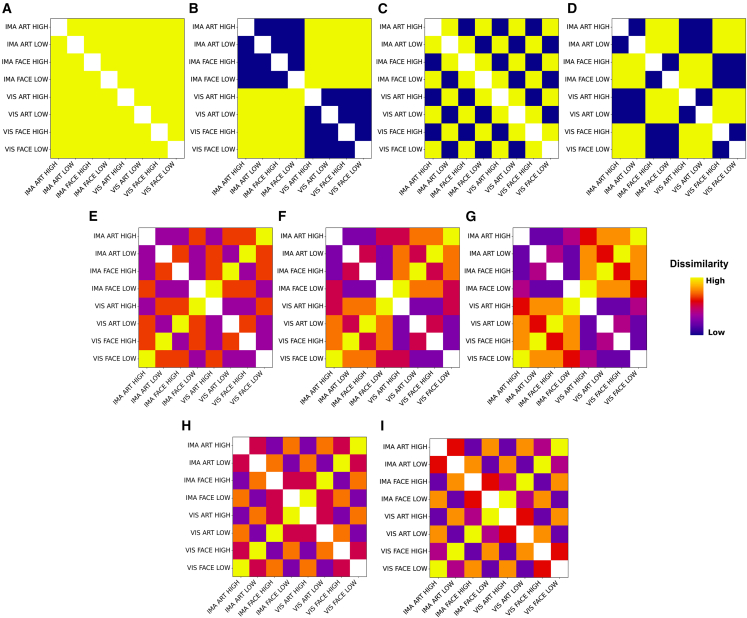
Candidate models (A) Independent model: predicts that all conditions are maximally dissimilar to each other. (B) Modality model: predicts that conditions across modalities are maximally dissimilar. (C) Experience model: predicts that conditions across elicited aesthetic experiences are maximally dissimilar. (D) Type model: predicts that conditions across stimulus types are maximally dissimilar. (E) Mod|Exp|Type model: combines equally the dissimilarity structure of modality, experience, and stimulus type. (F) Mod|(Exp|Type)/2 model: combines modality, experience, and stimulus type but downweighs experience and type by a factor of 2. (G) Mod|(Exp|Type)/3: combines modality, experience, and stimulus type but downweighs experience and type by a factor of 3. (H) Exp|(Mod|Type)/2: combines modality, experience, and stimulus type but downweighs modality and type by a factor of 2. (I) Exp|(Mod|Type)/3: combines modality, experience, and stimulus type but downweighs modality and type by a factor of 3.

To confirm that the neural patterns encoding the aesthetic experience are locally restricted within areas specialized in aesthetic processing while differences in modality are encoded more strongly across the entire brain, we performed a RSA across three levels of resolution; the whole brain; the reward system as defined by a meta-analytical mask using Neurosynth^48^; and two specific regions of interest (ROIs) within the striatum, that is, nucleus accumbens and caudate nucleus. The latter two ROIs were specifically chosen due to the consistent evidence in the literature that they are robustly involved in the anticipation as well as processing of aesthetic experiences.^40^^,^^41^^,^^42^ Ultimately, this approach enabled us to examine a common, implicit assumption; the effect of the external stimulation on the neural response is strongest at the whole brain level, while the subtler effects of the aesthetic experience are mainly encoded in highly specialized areas. Thus, at the whole brain level, we expected neural patterns to primarily reflect differences in stimulation modality since neural patterns would largely involve signals from primary visual areas and regions of the dorsal and ventral visual stream. However, when narrowing the focus to the reward network and even further to its constituents, we expected a shift in the neural signal to now largely reflect differences in the evoked aesthetic experience and only to a lesser extent other stimulus properties. Thus, depending on the level of interest, we hypothesized that the neural signal would be dominated by different aspects of the stimulation: at the whole brain, it would be dominated by differences in stimulation modality whereas at the more fine-grained level within the reward network, it would be dominated by differences in the evoked experiences. To investigate this assumption we used RSA, which assesses the dissimilarity of neural response patterns between conditions by creating neural representational dissimilarity matrices (RDMs) and comparing their similarity structure to theory-derived predictions about the geometry of evoked responses.^49^ To disentangle the influence of different aspects of the stimulation, such as modality of perception, stimulus type, and evoked aesthetic experience (high and low) on the recorded neural patterns, we tested 9 candidate models (see Figure 3). These models tested mainly five assumptions: (1) The neural patterns are completely independent of the stimulus properties (independent model). (2) Evoked neural patterns are solely driven by specific properties of the stimulus, that is, either by the modality of the stimulus, the experience the stimulus evoked, or the type of the stimulus (modality model; experience model; type model). (3) Evoked neural patterns are equally driven by stimulus properties (Mod|Exp|Type model). (4) Modality has a stronger influence on the neural signal compared to other stimulus properties. Models captured this assumption by downweighing the influence of evoked experience and type by a factor of 2 or 3 (Mod|(Exp|Type)/2 model; Mod|(Exp|Type)/3 model). (5) Experience has a stronger influence on the neural signal compared to other stimulus properties. Models captured this assumption by downweighing the influence of modality and type by a factor of 2 or 3 (Exp|(Mod|Type)/2 model; Exp|(Mod|Type)/3 model). Note that the latter two model types (4 and 5) were particularly designed to test H3, examining whether the contribution of stimulus properties to the neural signal varies across levels of resolution. Specifically, we expected model type 4 to perform better at the whole-brain level, while at the level of the reward network and its individual regions, we expected the evoked experience to play a more dominant role, as captured by model 5. Model performances were compared against the noise ceiling, which can be intuitively thought of as the performance that the true underlying data-generating model would achieve given the noise in the data. Thus, models that are indistinguishable from the noise ceiling can be viewed as adequately specified as they reach optimal performance. Additionally, at each level of resolution, we compared performances across models to examine whether certain models predict observed similarity patterns better than others. Notably, upon reviewer’s request, we additionally created a partial model solely combining aesthetic experience and stimulus type [Exp|Type] to investigate their combined influence on neural patterns; results are shown in the Supplemental Material (see Figure S1; Table S8).

**Figure 4:**
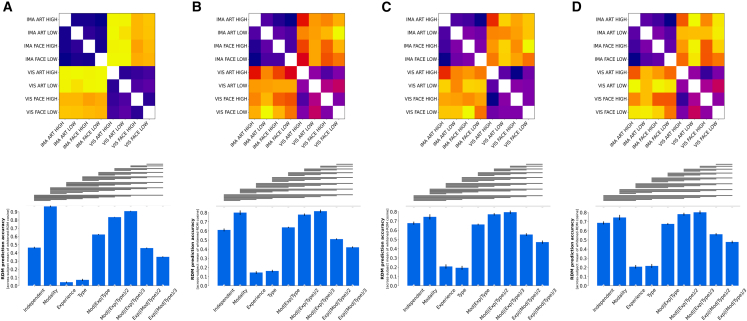
Performance of candidate models across brain regions for all participants (*n* = 30) (A) Model comparison for whole brain data. (B) Model comparison for reward network data. (C) Model comparison for nucleus accumbens data. (D) Model comparison for caudate nucleus data. Upper panels show across subject averaged neural RDMs. Lower panels show performance of candidate models across brain regions. Bars show means of whitened cosine similarity between candidate models and neural reference RDMs. Error bars indicate standard error of the mean (95% CI over 2000 bootstrap samples). Horizontal lines indicate significant differences in model performances (FDR q < 0.01). Gray dots on top depict significant differences from the noise ceiling (Bonferroni-corrected for 9 models). White dots at the bottom depict significant differences from 0. See also Table S9.

As expected, at the whole brain level, the Modality model—predicting that observed (dis)similarities are solely driven by differences in stimulation modality (perception vs. imagery)—outperformed all other candidate models while also reaching the noise ceiling, indicating that observed (dis)similarity patterns were best described by purely taking differences in modality into account (accuracy: 0.965 [+/− 0.010]) (see Figure 4). While this result aligns with our initial hypothesis that neural signals at the whole brain level would primarily encode differences in modality, the strength of this association is still surprising (for model performances see Table S9). Notably, even models that predicted just minor contributions from other stimulus properties, e.g., Mod|(Exp|Type)/3 model, performed significantly worse, underscoring the overwhelming influence of the difference in modality (perception vs. imagined) on the neural signal at this level.

Within the reward network, both the Modality model (accuracy: 0.801 [+/− 0.024]) and the Mod|(Exp|Type)/3 model (accuracy: 0.816 [+/− 0.016]) reached the noise ceiling, indicating that observed (dis)similarity patterns were still predominantly driven by differences in modality. Subsequent model comparisons did not result in a significant difference between the two models. Thus, despite anticipating a shift in the relative contribution of stimulus properties to the neural signal in favor of the evoked aesthetic experience (high vs. low), the signal within the reward network remains predominantly driven by modality. Even more striking is that only one of the winning models predicts that type and aesthetic experience are driving similarity patterns at this level. This indicates that within the reward network—a network of areas canonically associated with reward processing—the neural signal continues to be dominated by differences in modality, while the subjective aesthetic experience contributes at most 20 percent to the signal.

Focusing more closely on the constituents of the reward network, that is, zooming even further into the nucleus accumbens and the caudate nucleus, the Mod|(Exp|Type)/3 model not only reached the noise ceiling but also outperformed all other models (nucleus accumbens [accuracy: 0.794 [+/− 0.018])]; caudate nucleus [accuracy: 0.801 [+/− 0.019]). This suggests that as the level of resolution becomes more fine-grained within the reward network, neural patterns increasingly reflect the evoked aesthetic experience. Yet, contrary to our assumption, modality—not subjective aesthetic experience—remains the primary driving factor in nucleus accumbens and the caudate nucleus.

Taken together, these results showcase that even within highly specialized areas canonically associated with aesthetic processing neural (dis)similarities are still mainly driven by differences in modality and only to a much lesser extent by the evoked experience itself.

**Figure 5:**
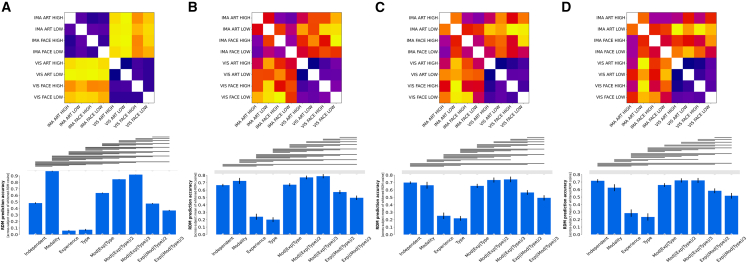
Subgroup analysis including only highly vivid trials for each participant (*n* = 11) (A) Whole brain results exclusively using highly vivid trials. (B) Reward network results exclusively using highly vivid trials. (C) Nucleus accumbens results exclusively using highly vivid trials. (D) Caudate nucleus results exclusively using highly vivid trials. Upper panels show across subject averaged neural RDMs. Lower panels show performance of candidate models across brain regions. Bars show means of whitened cosine similarity between candidate models and neural reference RDMs. Error bars indicate standard error of the mean (95% CI over 2000 bootstrap samples). Horizontal lines indicate significant differences in model performances (FDR q < 0.01). Gray dots indicate significant differences from the noise ceiling (Bonferroni-corrected for 9 models). White dots at the bottom depict significant differences from 0. See also Table S10.

To ensure that modality itself, rather than differences in vividness of the mental image, is driving observed similarity patterns, we conducted a subgroup analysis using only trials with the highest vividness ratings (6 and 7). Despite the reduction in sample size, the results remain largely comparable across the three levels of resolution (see Figure 5). The modality model continued to outperform all other models at the whole brain level (accuracy: 0.972 [+/− 0.005]) (see Table S10). Likewise, models that predicted stimulus type and experience had a small but significant impact on observed (dis)similarity patterns besides stimulus modality, i.e., Mod|(Exp|Type)/2 model and Mod|(Exp|Type)/3 model, performed exceptionally well within the reward network and its constituents (Mod|(Exp|Type)/2 model: reward network [accuracy: 0.771 [+/− 0.025]); nucleus accumbens (accuracy: 0.732 [+/− 0.035]); caudate nucleus (accuracy: 0.721 [+/− 0.028]); Mod|(Exp|Type)/3 model: reward network (accuracy: 0.787 [+/− 0.029]); nucleus accumbens (accuracy: 0.740 [+/− 0.039]); caudate nucleus (accuracy: 0.721 [+/− 0.034]). While the lower power from using exclusively highly vivid trials affected variance estimates and in turn model comparisons, the overall findings remain largely consistent with the main analysis. Thus, in light of these similar findings across the two analyses, it becomes apparent that aesthetic experiences generally are only marginally encoded in the neural patterns even within the reward network while the modality, in which the stimulus is originally perceived in, is dominantly driving neural dissimilarities across all levels of resolution.

## Discussion

This study tested the implicit assumption, prevalent within the field of neuroaesthetics, whether the aesthetic experience necessitates direct input into the sensory system by examining whether this complex and profound state could equally arise through purely internally generated representations. By presenting a diverse range of pre-rated aesthetic stimuli—artworks and faces—across sensory perception and mental imagery conditions, our study provides first conclusive, experimental-based evidence that the emergence of an aesthetic experience does not necessitate external sensory stimulation. The behavioral data clearly demonstrates that visual mental imagery is sufficient to elicit very similar aesthetic experiences to those evoked by visual perception when controlled for vividness. Interestingly, despite these overwhelming behavioral similarities, the neural signal in highly specialized areas canonically associated with aesthetic processing is only minimally driven by the subjective experience but rather dominated by differences in stimulation modality.

Across all tested facets of the aesthetic experience, visual perception evoked stronger aesthetic experiences than mental imagery. Yet, given that vividness plays a crucial role in shaping mental representations and naturally varies between trials,^17^ we suspected that these observed differences are driven by the variations in vividness of the mental images rather than the modality of presentation. When examining these trial-to-trial changes in vividness, the data clearly shows that the similarity in aesthetic ratings—that is, the similarity in subjective experiences—across visual perception and mental imagery is significantly affected by the vividness of the mental image. Thus, as mental representations more closely mirror those created by external perception, the resulting aesthetic experiences also become more similar. Notably, this effect is even present in participants specifically recruited for their average to high vividness abilities, suggesting that vividness might play a much stronger role in shaping the aesthetic experience in the general population. However, harnessing the advantages of the Bayesian framework, our results also evidently showed that for trials in which participants were able to clearly picture the presented stimulus, the two stimulation modalities elicited identical aesthetic experiences. This is in line with work by Dijkstra and colleagues who demonstrate that if the vividness of a mental image crosses a certain threshold, the induced experience becomes almost indistinguishable from the one induced by real-world events.^39^ In that respect, our findings reveal that mental imagery is not only potent enough to evoke complex second-order effects, such as aesthetic experiences but that these experiences—provided that mental images are vivid enough—become virtually indistinguishable from those elicited by visual perception.

Furthermore, using a diverse pool of aesthetic stimuli, the present study reveals that aesthetic experiences can reliably be evoked by a variety of stimulation types, man-made, culturally significant objects (artworks), and biological stimuli (faces), both during immediate sensory perception and visual mental imagery. However, it appears that man-made objects lead to a stronger aesthetic experience. This would indeed make sense given the notion that man-made, culturally significant stimuli are rich of contextual complexity, which can also particularly appeal to the specific cultural characteristics, expertise, and learned expectations of the respective viewer.^9^^,^^50^ Previous studies have continuously indicated that the more context and complex information provided for a stimulus, the higher was the aesthetic response.^51^^,^^52^^,^^53^^,^^54^^,^^55^^,^^56^^,^^57^^,^^58^^,^^59^ In addition, prior research has demonstrated that both shared and individual (idiosyncratic) taste play a crucial role in shaping an aesthetic experience. However, individual taste has a greater potential to elicit strong aesthetic moments such as awe and being moved. Since aesthetic judgments of artworks, compared to faces, are significantly more affected by idiosyncratic taste,^60^ it is to be expected that artworks consistently elicited more profound experiences than faces. Nonetheless, the aesthetic experience can reliably emerge—independently of direct external sensory perception—across a diverse range of visual stimuli, as our behavioral data unambiguously demonstrates, even though the type of stimulation has a significant influence on the strength of the evoked experience.

Two key implications arise from the results of the RSAs. First, stimulus modality is clearly driving observed (dis)similarities across conditions. Among all three levels of resolution, the modality model, which exclusively assumes modality is driving observed dissimilarity patterns, performed exceptionally well compared to other candidate models even reaching the noise ceiling at the whole brain and reward network level. The noise ceiling indicates that observed differences were explained just as accurately by purely considering the conceptual (dis)similarities across direct external sensory perception and visual mental imagery as they could have been by the underlying true (albeit unknown) data-generating model. Even though, we hypothesized that the neural signal would primarily encode differences in modality at the whole brain level, this basic model outperformed even models which were taking only minor contributions of stimulus type and the aesthetic experience into account. The fact that this association was also evident in the subgroup analysis, using exclusively highly vivid trials, suggests that the difference between visual perception and visual imagery dominates the neural signal (despite studies showing that similarity of neural patterns increases with vividness^17^^,^^61^). Even though the whole brain mask included also a variety of regions typically linked to stimulus recognition (e.g., the ventral visual stream^62^) and aesthetic processing (e.g., the reward network^40^^,^^41^^,^^42^), neither stimulus type nor the evoked aesthetic experience significantly influenced the neural signal at this broad level of analysis. But more surprisingly is the fact that the dominance of modality persists even when zooming into highly specialized areas for reward processing such as the reward network and the nucleus accumbens and caudate nucleus. This is contrary to our hypothesis (H3) predicting that the neural signal at the whole brain level would be dominated by modality but that this pattern would reverse within areas highly specialized in reward processing. In contrast to this common notion, however, our study revealed that the neural signal predominantly reflects whether a stimulus was externally or internally generated—even in areas highly specialized in aesthetic processing. Taken together, neural patterns across all levels of resolution did quite substantially encode differences in stimulation modality rather than evoked experience, despite the finding that behaviorally aesthetic experiences were indistinguishable across modalities for highly vivid trials.

Second, nonetheless, the more the regions were restrained to areas associated with aesthetic experiences, the more were neural (dis)similarity patterns encoding differences in evoked experiences and stimulus type besides changes in stimulus modality. In other words, within areas closely associated with aesthetic experiences such as the caudate nucleus and the nucleus accumbens, the Mod|(Exp|Type)/3 model, which was taking the experience and type of stimulus into account (albeit three times less significant as modality), not only outperformed all other models but also reached the noise ceiling indicating that this model perfectly described the recorded patterns. Focusing on the relative contribution of the subjective experience to the neural signal of about 20 percent, it is remarkable that despite its name, neural patterns within the reward network only encode evoked experiences to a quite small extent. The same pattern emerged when further zooming into the reward network, that is, the nucleus accumbens and the caudate nucleus—two areas reliably and continuously associated with aesthetic processing. Hence, although visual mental imagery is capable of generating complex and profound aesthetic experiences almost indistinguishable from those induced by visual perception, the neural signal underlying these very similar experiences differs substantially between the two modalities.

In conclusion, the present study provides first experimental-based proof that aesthetic experiences can arise solely through visual mental imagery without the need of immediate external sensory perception. Even though vividness predicts the similarity between evoked experiences across modalities, highly vivid and precise internally generated representations elicit indistinguishable experiences from visual perception. Finally, contrary to our initial hypothesis, peak aesthetic experiences are not dominantly encoded in regions primarily associated with aesthetic processing. Rather, neural patterns are primarily driven by stimulation modality and only to a marginal extent by stimulus type and aesthetic experience. Thus, despite the fact that the subjective quality of the aesthetic experience does not appear to be affected, the stimulation modality provides a unique contribution to its underlying neural pattern. Taken together, this provides a new perspective on how the external world and an individual’s internal processes shape the emergence of experiences, particularly aesthetic ones. While the modality of stimulation plays a significant role in how the brain processes information at all levels, the resulting aesthetic experiences can be remarkably similar, making them largely independent of the origin of the experience-inducing stimulus.

### Limitations of the study

Despite efforts of the current study to recover unbiased similarity estimates by distantly spacing stimulus presentations, not all sources of bias could be fully eliminated. In general, it is advised to use crossnobis dissimilarity estimates if more than one run is recorded as they recover similarity structures more accurately.^63^ Unfortunately, the subjective nature of the aesthetic task resulted in a majority of participants having a condition (e.g., a high aesthetic experience in response to an imagined facial stimulus) only being present in one run, rendering cross-validated similarity measures futile. Thus, if peak subjective experiences are of scientific interest, future research needs to balance methodological constraints with stimulus novelty and feasibility. Nonetheless, given the high similarity between the results of the complete dataset and the subset including only trials that had a high vividness rating, we are confident that the winning theory-derived candidate models adequately describe the recorded patterns. Furthermore, we acknowledge that it might have been possible that participants retained previously seen visual stimuli in their visual working memory rather than reimagining them. We deem this rather unlikely as the two modality conditions were spaced roughly 23 s apart in which several questions regarding the evoked aesthetic experience have been assessed. Lastly, a potential limitation of the current design is the possibility that ratings of the aesthetic experience persisted from the visual perception to the mental imagery condition. However, given the variability in ratings—sometimes leading to extremely different behavioral experiences across modalities—we consider this as unlikely as well. Nonetheless, future studies might aim to better separate the rating phases between the two modalities. Achieving this, would likely require training participants to memorize stimuli and recall them when cued, making it difficult to examine a large set of complex, novel, and naturalistic stimuli, as has been implemented in the present study.

## Resource availability

### Lead contact

Requests for further information and resources should be directed to and will be fulfilled by the lead contact, Maximilian Kathofer (maximilian.kathofer@univie.ac.at).

### Materials availability

This study did not generate new materials.

### Data and code availability

• All behavioral data and beta estimates have been deposited at OSF: https://osf.io/apjud/?view_only=13f6a8e9adbb485385306b7204c31456 and are publicly available as of the date of publication.
• All original code has been deposited at OSF: https://osf.io/apjud/?view_only=13f6a8e9adbb485385306b7204c31456 and is publicly available as of the date of publication.
• All stimulus materials can be freely downloaded for scientific use.
• Chicago Face database: https://www.chicagofaces.org/.
• Face Research Lab London Set: https://figshare.com/articles/dataset/Face_Research_Lab_London_Set/5047666.
• Vienna Art Picture System: https://osf.io/a7xcr/.

## Acknowledgments

This research was funded in whole or in part by the Austrian Science Fund (FWF) 10.55776/CM11. M.K. is funded by 10.13039/501100013699BMBWF and 10.13039/501100005203OeAD-GmbH MMC-2023-06988. We want to thank Marc Esmeyer and Marius Schaffer for their fundamental work in helping to create stimulus material, in data acquisition, and in implementing the pilot study. The smiling person icon in the graphical abstract was created by iconcheese from the Noun Project.

## Author contributions

Conceptualization, H.L. and J.S.C.; data collection, M.K. and J.S.C.; formal analysis, M.K. and J.S.C.; writing – original draft, M.K. and J.S.C.; writing – review and editing, H.L., C.L., J.S.C., and M.K.; resources, H.L., J.S.C., and C.L.; supervision, J.S.C.; visualization, M.K.

## Declaration of interests

The authors declare no competing interests.

## Declaration of generative AI and AI-assisted technologies

During the preparation of this work the author(s) used ChatGPT and Grammarly in order to check spelling, grammar, and phrasing. After using this tool/service, the author(s) reviewed and edited the content as needed and take(s) full responsibility for the content of the publication.

## STAR★Methods

### Key resources table

**Table:** 

REAGENT or RESOURCE	SOURCE	IDENTIFIER
**Deposited data**		

All code, stimuli, beta maps and behavioral data	Center for Open Science https://www.cos.io/	https://osf.io/apjud/?view_only=13f6a8e9adbb485385306b7204c31456

**Software and algorithms**		

R 4.4.0	The R Project for Statistical Computing	https://www.r-project.org/
MATLAB 2019a	Mathworks, Inc, MA, USA	https://www.mathworks.com/products/matlab.html
Nipype	Gorgolewski et al.^64^	https://github.com/nipy/nipype
ANTs	Advanced Normalization Tools	https://github.com/ANTsX/ANTs
FSL	Jenkinson et al.^65^	https://fsl.fmrib.ox.ac.uk/fsldownloads_registration/
rsatoolbox	RSAtoolbox Development Group^66^	https://rsatoolbox.readthedocs.io/en/stable/
synthstrip	Hoopes et al.^67^	https://surfer.nmr.mgh.harvard.edu/docs/synthstrip/
ICA-AROMA	Pruim et al.^68^	https://github.com/maartenmennes/ICA-AROMA
NeuroSynth	Yarkoni et al.^48^	https://neurosynth.org/
PsychoPy	Pierce et al.^69^	https://www.psychopy.org/
G∗Power	Faul et al.^70^	https://www.psychologie.hhu.de/arbeitsgruppen/allgemeine-psychologie-und-arbeitspsychologie/gpower

### Experimental model and study participant details

Participants were recruited at the campus of the University of Vienna and the central Viennese metropolitan area using flyers. To control for the effects of vividness on the aesthetic experience, participants were preselected using the Vividness of Visual Imagery Questionnaire (VVIQ)^20^ to exclude all with low visual imagery abilities. The original VVIQ (see Data S1) is a 16-item self-report questionnaire assessing the ability to create clear mental images, yet, for screening efficiency and given its unidimensional structure^71^ in conjunction with its extremely high internal consistency,^72^ we only used 8 of its items. Typically, a 32-point cut-off is applied to identify individuals with extremely low imagery ability, a condition known as aphantasia.^73^ For our shortened version, we implemented a more conservative proportional threshold of 25 instead of 16 points to ensure that participants did not exhibit very low imagery abilities. The translation of items into German was implemented by an English teacher and verified by a bilingual researcher. Items were adapted to fulfill the University’s gender policy and to improve comprehensibility (see Data S2). To additionally ensure that participants experience at least average emotional and physiological responses to aesthetic stimuli, we used a slightly adapted version of the Aesthetic Experience Scale (AES)^74^ (see Data S3), which measures the frequency and strength of aesthetic experiences. Translation to German and then back to English was implemented by three native speakers in each language respectively. Slight modifications were made to tailor questions to entail favorite art domains and frequency of exposure to these domains (see Data S4). Participants needed a minimum sum score of 35 on the AES and 25 on the VVIQ to qualify for the study, ensuring the exclusion of individuals with low aesthetic and imagery capabilities. Since this study was not interested in investigating the effect of vividness across the whole population, these cut-offs ensured that imagery was vivid and comparisons between modalities were safeguarded against potential biases stemming from participants’ inability to mentally imagine stimuli, and thus, low ability to have aesthetic experiences in the first place. Additionally, participants had to meet the following inclusion criteria: at least 18 years of age, fluency in German, right-handedness, MRI compatibility, and normal or corrected to normal vision. Exclusion criteria included history of psychiatric and psychological disorders, medication affecting mood, (possible) pregnancy, and claustrophobia.

One participant was excluded during the study because of previously not declared mood-affecting medication and one other because of a technical issue terminating the scan session during recording. The final sample consisted of 34 participants (21 females, 1 diverse, 12 males), with a mean age of 23.48 years (SD = 4.01), all of whom provided informed consent. Thirty-two were native German speakers and the other two participants reported high proficiency in German. The study was approved by the ethics committee of the University of Vienna (Reference Number: 00636).

### Method details

#### Procedure

To assess the neural representation of hedonic experiences in response to perception and imagery, participants had to complete an aesthetic task while functional magnetic resonance imaging (fMRI) data were collected. Commonly in aesthetic research, definitions of assessed concepts, such as being moved, are not adequately provided as they are assumed to be universally known. This is not only unwarranted, but an in-house pre-study (data not shown) demonstrated that laypeople differ quite considerably in their definitions of these concepts, which is why we provided clear instructions and definitions of the rating dimensions prior to the aesthetic task. To further mitigate any semantic misconceptions, participants had to pass comprehension questions assessing their understanding of the individual concepts (see Data S5).

#### Sample size calculation

*A-priori* sample-size calculations were based on a study with similar design^75^ for a within-subject main effect of at least Cohen’s *d* = 0.25 using G∗Power^70^ (alpha: 0.05; power: 0.80).

#### Stimulus selection

The stimulus set contained both representational artworks^40^^,^^41^^,^^60^^,^^76^ and faces^77^^,^^78^^,^^79^ as they are the most prevalent stimulation types used in aesthetic research. Additionally, using these two very distinct types of stimulation enabled us to examine whether our findings generalize across different types of stimuli, that is, either biologically or culturally significant (man-made).

A pre-study was conducted to maximize the likelihood of face stimuli eliciting strong aesthetic experiences. Seventeen participants (female: 7; *M*: 29.7; *SD* = 11.3) rated a predefined set of 56 faces extracted from the Chicago Face Database,^80^ the Face Research London Set,^81^ and the database of Schacht.^82^ Using Adobe Photoshop, unnatural color gradings were edited and picture backgrounds were standardized. Faces were rated along the dimensions: beauty, arousal, valence, aesthetically moving, interest, and liking. The final set of 20 stimuli (12 male & 8 female faces) was chosen in such a way that half of the set elicited neutral and the other half maximal aesthetic experiences. We believe that the unbalanced distribution of gender does not affect our research question since we are not interested in investigating whether faces across gender induce a distinct brain response but rather use these stimuli to induce an aesthetic experience. The selected stimuli were rated the highest independent of gender and sexuality.

Using the pre-rated “Viennese Art Picture System” database (VAPS),^83^ which contains 999 artworks of various styles, we extracted 20 highly beautiful and neutral representational paintings. Half of the selected stimuli elicited high mean beauty ratings – usually above 90 out of 100 points – with low variance while the other half was characterized by ratings below 50. Importantly, even though this study primarily focuses on the strong aesthetic experience of being moved, artworks were not extracted depending on their moving ratings from the database since participants initially rating these artworks were not given a sufficient definition of this rather complex concept *a priori*, and we suspected high interrater variance due to semantical misconception.

#### Aesthetic task

The final stimulus set consisted of 20 faces and 20 representational artworks, which were split randomly across 4 runs. PsychoPy was used for stimulus presentation.^69^ Following a jittered fixation cross (3–5 s), a stimulus was presented for 15 s. Presentation length was chosen based on results from Belfi and colleagues,^41^ showing that areas important for reward processing (nucleus accumbens and caudate nucleus) only respond to aesthetic processing during longer trial durations (5–15 s). Thereafter, participants rated the evoked experience along the dimensions 1) beauty (“How beautiful was the image”), 2) aesthetically moving (“How aesthetically moving was the image”), and 3) valence (“How pleasurable was the image”) on a 7-point Likert scale. Given that pleasure and beauty are commonly assessed aesthetic dimensions, despite a lack of precise definitions in previous studies, we included them to explore whether induced effects differ across these aesthetic dimensions as well, even though the main focus of this study was the profound state of being moved. The order of questions was randomized across trials, and participants had 6 s to answer each question. Subsequently, participants were shown a symbol for 5 s which prompted the participants to close their eyes and prepare for the visual imagery trial, in which they had 15 s to imagine the previously encountered stimulus as vividly as possible. The beginning and end of this time interval were marked by a sinus tone (1 s), so participants knew when to start imagining and when to open their eyes again. Participants rated the mental image alongside the dimensions described above as well as its vividness (“How vivid was your mental image”). Each run lasted approximately 13–14 min.

Due to a technical issue, participants 3–11 were only presented 36 of the 40 stimuli, which alternated randomly between participants. Furthermore, compared to all other subjects, the task for subjects 1 and 2 was not split in 4 equal runs but 1 and 2 runs, respectively. Due to the subjective nature of the experiment, elicited subjective experiences generally resulted in some of the 8 conditions being only present in one of the runs, rendering crossnobis similarity estimates futile. Thus, we opted for a priori-defined across-runs averaged beta estimates and consequently could also use the data of subject 1 and 2 without biasing the estimates.

#### MRI data acquisition

Each block was recorded in a separate fMRI run. Functional data were acquired on a Siemens MAGNETOM Skyra 3T scanner with a sequentially increasing multiband 4 sequence (voxel size: 2.2^3^ mm; TR: 0.915 s; TE: 34 ms; FA: 55°, FoV: 210 mm, FoV phase: 100%) and a 32-channel head coil. To aid magnetic inhomogeneity correction, field maps were recorded at the end of each session. T1-weighted structural scans (MPRAGE; voxel size: 0.94^3^; TR: 2.3 s; TE: 2.29 ms; TI: 900 ms; FA: 8°; FoV: 240 mm, FoV phase: 100%) were acquired for each participant.

#### MRI data preprocessing

##### Structural

Using nipype,^64^ T1 scans were bias field corrected using the well-known N4ITK bias field correction algorithm due to its superior performance compared to its predecessor^84^ and its subjective better performance than FSL FAST. Then, the images were brain extracted using Synthstrip as it outperforms other commonly used algorithms once with the –no-csf flag and once with the flags –no-csf and -b 2 (since those parameter settings usually yielded the best extractions).^67^ Results were visually inspected and the best extractions were chosen for further analysis.

##### Field maps

Using custom MATLAB^85^ scripts, magnitude images were reoriented, bias field corrected (N4ITK), brain extracted (Synthstrip), and eroded, so images only contained brain voxels following FSL recommendations. Phase images were prepared for their use within the FEAT pipeline using fsl_prepare_fieldmaps.

##### Functional

Using the FSL FEAT^65^ pipeline, images were B0-unwarped (FUGUE), realigned to the middle volume (McFLIRT), coregistered to the structural scan using Boundary Based Registration (BBR) as it is more accurate than other commonly used methods (that is, correlation and mutual information)^86^ and spatially smoothed using a 6mm FWHM (SUSAN). A 6 mm kernel was chosen since ICA-AROMA^68^ is validated with the same settings. Images were registered to MNI 2mm standard space using the default settings of antsRegistrationSyN.sh as it outperforms other commonly used nonlinear algorithms.^87^^,^^88^ To further mitigate potential bias introduced by artifacts, we used ICA-AROMA in its default settings (non-aggressive) since it is superior to other pipelines in balancing efficiency with data loss.^89^ High-pass filtering was performed using fslmaths’ -bptf flag with a sigma of 44.08 (82 s), which corresponds to one full trial cycle.

#### Excessive motion

Since the efficiency of ICA-AROMA decreases for high-motion data, we additionally excluded whole datasets based on the recommendations of Parkes et al.^89^ exceeding a threshold of 0.2 mean framewise displacement (FD), having more than 20% outliers, or a maximal FD of 5 mm. Based on these criteria, one participant was excluded.

#### Regions of interest

To confirm that the neural patterns encoding the aesthetic experience are locally restricted within areas specialized in aesthetic processing in contrast to differences in modality which are encoded in the neural patterns across the entire brain, we have chosen three levels of resolution: (1) the whole brain; (2) the whole reward system as defined by a meta-analytical mask using the term ‘reward’ in Neurosynth^48^ (https://www.neurosynth.org/analyses/terms/reward/, association test map thresholded at FDR <0.01); (3) two specific ROIs within the striatum, that is, nucleus accumbens and caudate nucleus. These two ROIs were chosen as they are consistently implicated in the anticipation as well as the processing of aesthetic experiences.^40^^,^^41^^,^^42^

### Quantification and statistical analysis

#### Missing data

Except for the 4 missing stimuli for participants 3–11, which alternated randomly between partitions of the whole stimulus set for each subject, missingness only occurred in dependent variables, i.e., judgments of each evoked experience. Overall, less than 2.6% of recorded responses were missing (see Figure S2). In general, statistical tests can only detect whether data are missing completely at random (MCAR), thus not biasing the statistical analysis, or missing at random (MAR), that is, missingness depends on unobserved variables and consequently might substantially bias estimates. Using the R package mice^90^ and its mcar function, we found no conclusive evidence that the data might be MAR as our data were not multivariate normally distributed (Anderson-Darling: median *T* = 8.10; median *p* = 0.25), and thus, the Hawkin’s test could not be interpreted. As statistical assessment of MAR is often regarded suboptimal,^91^ we reasoned that missingness might most probably be due to the restricted 6-s time window to answer each question. Judging from the almost uniform distribution of missing values across response dimensions and no clear patterns emerging between participants, we did not suspect hidden variables to cause missingness. In summary, we conclude that the missing values were truly MCAR. In line with the fact that the proportion of missing data remained below the commonly cited 5% threshold,^92^ we opted for a complete case analysis, which in this case ensures unbiased estimates.

#### Hierarchical Bayesian ordinal regression to compare experiences across conditions

To assess whether aesthetic experiences can occur without external sensory stimulation and whether this affects the quality of the experience (H1), we tested whether ratings of aesthetic experiences differ between stimulation modalities (visual perception & visual imagery) and stimulation type (face & artwork). We opted for a hierarchical Bayesian ordinal regression using the brms package^93^ since it takes both the nestedness and the nonnormal distribution of responses into account. In such cases, it is beneficial to model Likert-scale data as truly ordinal as studies have shown that modeling Likert-scale responses instead as metric increases both Type 1 and 2 errors and potentially leads to inverted effect sizes.^94^ Prior to modeling, we assessed which link function [probit, logit, cloglog, and cauchit] and threshold [flexible and equidistant] fitted our data best by creating null models with a series of possible configurations using the resulting log likelihood of each model as benchmark for model fit. Following the recommendations of Bürkner & Vuorre,^95^ we used leave-one-out cross-validation to systematically compare potential models containing various combinations of category-specific effects, included regressors and hierarchical effects. As initially expected, the winning cumulative model with a probit link function contained regressors for stimulus type (face & artwork) and modality (visual perception & mental imagery), and random intercepts for both participants (ID) and each stimulus (stimulus_ID) (see Table S11). Weakly informative priors were used for stimulus type and modality [normal: 0,1]^96^ and default brms priors for all other parameters [student t: 3,0,2.5]. All reported models converged and posterior predictive checks assessed adequate data description.

#### Hierarchical Bayesian linear regression to probe vividness

To assess whether evoked experiences become more similar between stimulation modalities depending on the vividness of the mental image (H2), we calculated an absolute similarity score between responses across modalities by subtracting each individual rating in the imagery condition from its visual counterpart, squaring it and then adding them together for each individual stimulus. This resulted in a vector ranging from 0 (perfect similarity) to 48 (highest observed dissimilarity). The Bayesian linear model used a student distribution and contained a regressor for vividness and a random intercept for each participant to account for the hierarchical structure of the data. Parameter estimates were standardized using the parameters package.^97^

#### Region of practical equivalence testing using Bayes Factors

To examine whether evoked aesthetic experiences across stimulation modalities result in equal subjective experiences (H1) - if controlled for vividness - we performed a region of practical equivalence test using the bayestestR package^98^ and assessed significance using the Bayes Factor. The resulting Bayes Factor indicates whether the posterior distribution of a parameter estimate has shifted away or toward a region of effect sizes [-0.1–0.1] typically considered as negligible.^45^^,^^46^ For this analysis, we only used trials with the highest vividness ratings (6 and 7), performed hierarchical ordinal Bayesian regressions using the brms package and then calculated the Bayes Factor for the modality regressor using the bf_rope function of bayestestR. The models included main regressors for stimulus modality and type and random intercepts for each participant and stimulus.

#### Beta estimates

Here, we focus on the profound emotional state of being moved as the main concept of interest as it describes a deep emotional response to aesthetic stimuli. To compare high and low aesthetic experiences across the range of different stimulation modalities and types, we excluded trials with average ratings (4) and then split moving responses into high and low experiences.

First, a standard GLM in FSL was constructed convolving stimulus onsets and durations with a gamma response function to obtain beta estimates for all task regressors of interest. The GLM contained separate regressors for each unique combination of the three conditions, that is, experience (high and low), modality (perception and imagery), and type (art and face): high perception art (HPA), low perception art (LPA), high perception face (HPF), low perception face (LPF), high imagery art (HIA), low imagery art (LIA), high imagery face (HIF), and low imagery face (LIF). Furthermore, to account for residual variance of the task regressors temporal derivatives were added to the model. Additionally, a separate regressor modeling question blocks and trials with missing values was added to the GLM. The fMRI time series was pre-whitened within FSL FEAT to account for temporal autocorrelation using an AR(1) model. Due to the subjective nature of evoked experiences resulting in the absence of certain conditions within specific runs for a given participant, empty regressors were included in such cases to maintain consistent degrees of freedom across runs. The resulting run-wise beta maps were averaged for each participant using a fixed-effects model.

#### Representational similarity analysis

Representational similarity analysis (RSA) enables the investigation of the neural representational structure of a stimulus across multiple dimensions.^99^ More specifically, RSA can be used to investigate how and if different aspects of a stimulus such as modality of perception, stimulus type, and experience are represented within a given neural pattern. To do so, RSA measures how dissimilar neural response patterns between conditions are by creating neural representational dissimilarity matrices (RDMs) and comparing them to theory-derived predictions about the geometry of evoked responses.^49^ Here, we probe 9 candidate models: 1.) Independent model: conditions are maximal dissimilar to each other, meaning the neural patterns are completely independent from the stimulus properties; 2.) Modality model: neural similarity patterns are solely driven by differences in modality, meaning that observed patterns are similar within the same and dissimilar across stimulus modalities regardless of stimulus type and evoked experience (external sensory perception vs. mental imagery); 3.) Experience model: neural similarity patterns are solely driven by differences in experience, meaning that observed patterns are similar within the same and dissimilar across evoked experiences regardless of stimulus modality and type (low vs. high); 4.) Type model: neural similarity patterns are solely driven by differences in stimulus type, meaning that observed patterns are similar within the same and dissimilar across stimulus type regardless of stimulus modality and evoked experience (faces vs. artworks); 5.) Mod|Exp|Type: this model is a combination of the three previous models and takes the similarity within experience, modality, and type into account, meaning neural patterns are equally driven by modality, experience, and type of stimulus; 6.) Mod|(Exp|Type)/2 model: This model predicts that the brain primarily encodes the initial modality of a stimulus, as it needs to track where a stimulus originated from – it still combines modality, type, and experience but reduces the influence of experience and type on the similarity of neural patterns by half, meaning differences in modality have 2 times more influence on neural patterns than experience or type; 7.) Mod|(Exp|Type)/3 model: similar to model 6, this model combines the similarity structure of stimulus modality, type, and experience, however, downweighs the latter two contributions by a factor of 3, meaning differences in modality have 3 times more influence on neural patterns than differences in experience or type; 8.) Exp|(Mod|Type)/2 model: this model predicts that the brain primarily encodes the strength of the experience induced by a stimulus – it still combines modality, type, and experience but reduces the influence of modality and type on the similarity of neural patterns by half, meaning differences in experience have 2 times more influence on neural patterns than modality or type; 9.) Exp|(Mod|Type)/3 model): similar to model 8, this model combines the similarity structure of stimulus modality, type, and experience, however, downweighs the former two contributions by a factor of 3, meaning differences in experience have 3 times more influence on neural patterns than modality or type.

Three participants were excluded from further analysis because they did not have responses in all conditions across the recorded runs.

The RSA analysis was performed using the Python Representational Similarity Analysis toolbox.^66^ First, we z-scored the across-runs averaged beta estimates and created neural RDMs using squared Euclidian distances for each participant.^100^ Neural RDMs were constrained to the aforementioned ROIs to examine whether the representational structure changes across different processing regions and levels of resolution. Next, we calculated the whitened cosine similarity between neural RDMs and the 9 candidate models.^101^ Within each ROI, the 9 candidate models were compared against 0 and the lower bound of the noise ceiling. Intuitively, the noise ceiling is a measurement of how good the true data generating model would perform if stimulus responses were fixed and would only differ in noise. Thus, comparing candidate models to the noise ceiling indicates whether they are appropriately specified (no significant difference) or lack specification (significant difference).^102^ Additionally, candidate models were compared against each other across all ROIs using two-tailed t-tests with FDR, q < 0.01. To calculate stable uncertainty estimates and compare model performances, we randomly sampled subjects 2000 times, which is twice the recommended sampling rate.^66^
