# Supplementary material for: Aesthetic experiences across visual perception and mental imagery: Behaviorally indistinguishable, neurally distinct
Source: iScience. 2025 May 5;28(6):112588. doi: 10.1016/j.isci.2025.112588 (PMC12148595; doi:10.1016/j.isci.2025.112588)
Supplement: Document S1. Figures S1 and S2, Tables S1–S11, and Data S1–S5 [file mmc1.pdf]

iScience, Volume *28*

Supplemental information

Aesthetic experiences across visual perception and mental imagery: Behaviorally indistinguishable, neurally distinct

Maximilian Kathofer, Claus Lamm, Helmut Leder, and Julia Sophia Crone

Representational Similarity Analysis: partial model

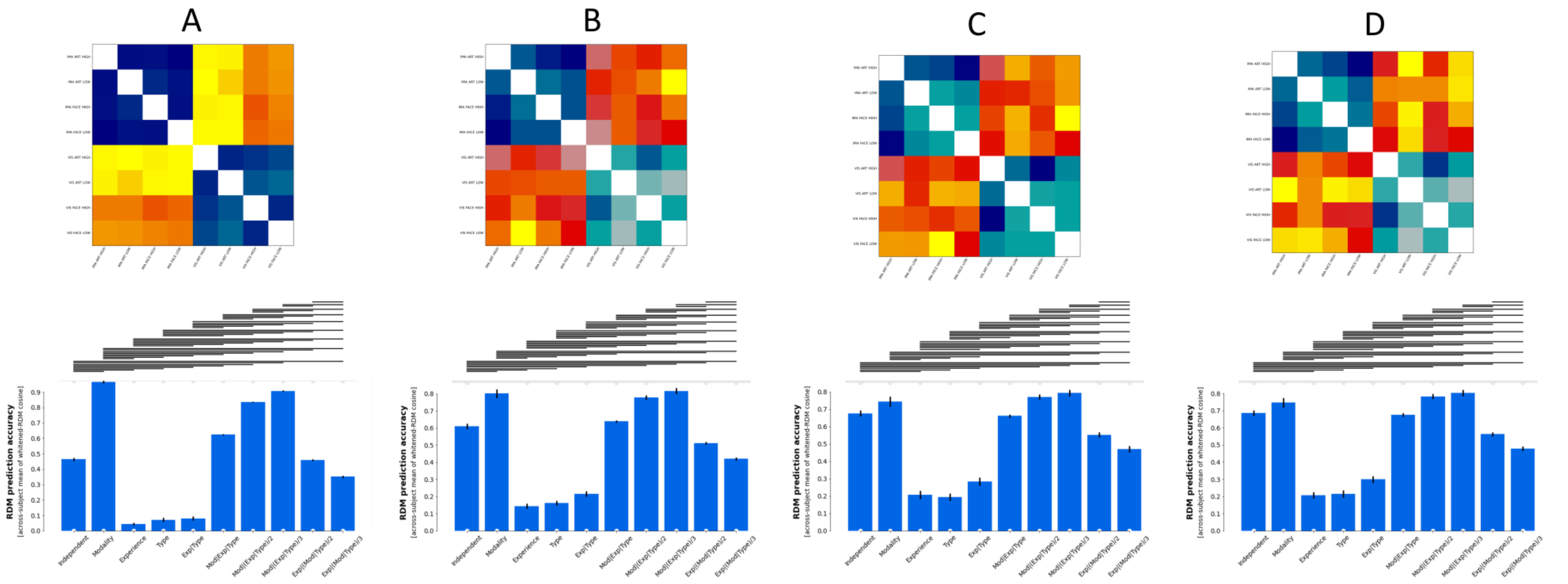

Figure S1. Performance of candidate models across brain regions for all participants (n = 30). (A) Model comparison for whole brain data. (B) Model comparison for reward network data. (C) Model comparison for nucleus accumbens data. (D) Model comparison for caudate nucleus data. Upper panels show across subject averaged neural RDMs. Lower panels show performance of candidate models across brain regions. Bars show means of whitened cosine similarity between candidate models and neural reference RDMs. Error bars indicate standard error of the mean (95% CI over 2000 bootstrap samples). Horizontal lines indicate significant differences in model performances (FDR q < 0.01). Grey dots on top depict significant differences from the noise ceiling (Bonferroni-corrected for 10 models). White dots at the bottom depict significant differences from 0.

Missing data

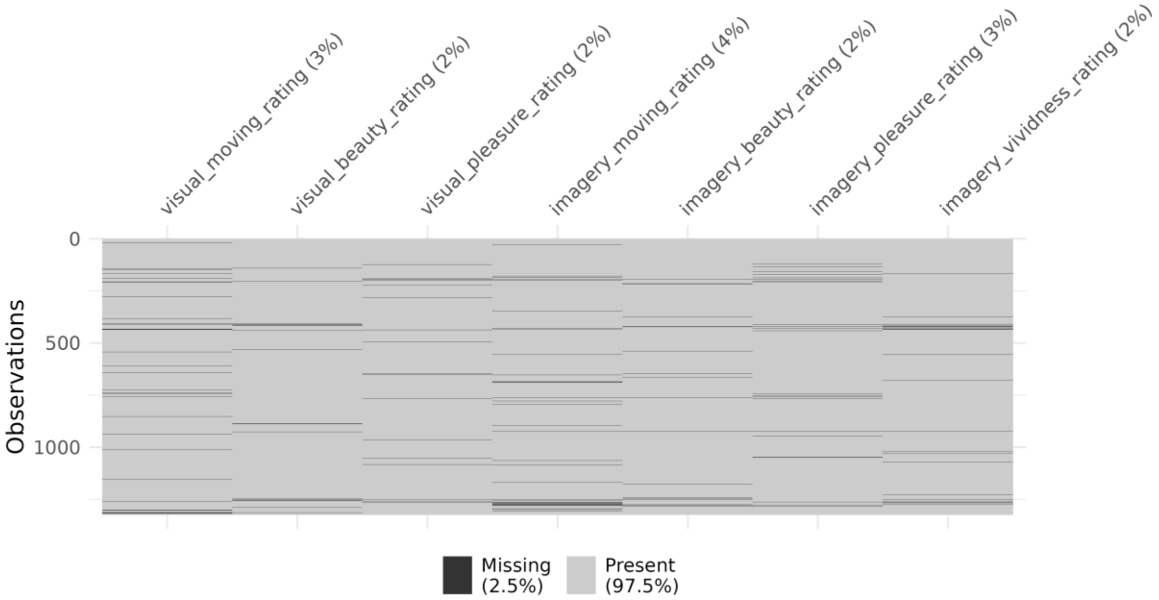

Figure S2. Missing data of response variables aggregated across participants. Data was plotted using the naniar package [S1].

Hierarchical ordinal Bayesian analyses of aesthetic facets for all trials

Moving:

Predictors	Estimate	Est.Error	l-95% CI	u-95% CI	Rhat	Bulk_ESS	Tail_ESS
**Intercept[1]**	-2.27	0.14	-2.55	-2.01	1.00	1678	3073
**Intercept[2]**	-1.34	0.13	-1.60	-1.08	1.00	1626	2807
**Intercept[3]**	-0.61	0.13	-0.87	-0.35	1.00	1611	2853
**Intercept[4]**	-0.15	0.13	-0.41	0.11	1.00	1602	2741
**Intercept[5]**	0.74	0.13	0.48	0.99	1.00	1627	2746
**Intercept[6]**	1.78	0.14	1.51	2.05	1.00	1787	3420
**Modality: Imagery**	-0.29	0.04	-0.37	-0.21	1.00	13091	7149
**Type: Face**	-0.56	0.11	-0.77	-0.35	1.00	2905	4787
**Random effects**							
**ID**	0.60	0.08	0.47	0.79	1.00	2064	3786
**Image_ID**	0.31	0.04	0.23	0.40	1.00	2833	3830

Table S1. Results of the hierarchical ordinal regression for moving ratings using all trials. Readers are reminded that estimates shown are standardized as they represent beta estimates on the latent standardized normal scale. Intercepts denote partitions of the latent scale to derive cumulative probabilities [S2]. Est.Error = estimation error. CI = Credible Interval. ESS = effective sample size. ID = random intercept for each participant (n=34). Image_ID = random intercept for each stimulus (n=40). Observations = 2553.

Beauty:

Predictors	Estimate	Est.Error	l-95% CI	u-95% CI	Rhat	Bulk_ESS	Tail_ESS
**Intercept[1]**	-2.48	0.14	-2.76	-2.21	1.00	1387	2596
**Intercept[2]**	-1.58	0.13	-1.84	-1.32	1.00	1296	2381
**Intercept[3]**	-0.95	0.13	-1.21	-0.69	1.00	1293	2374
**Intercept[4]**	-0.45	0.13	-0.71	-0.19	1.00	1273	2281
**Intercept[5]**	0.46	0.13	0.19	0.71	1.00	1262	2247
**Intercept[6]**	1.44	0.14	1.17	1,70	1.00	1367	2189
**Modality: Imagery**	-0.28	0.04	-0.37	-0.20	1.00	11821	6478
**Type: Face**	-0.43	0.15	-0.71	-0.13	1.00	1588	2736
**Random effects**							
**ID**	0.46	0.07	0.35	0.61	1.00	1927	3499
**Image_ID**	0.44	0.06	0.34	0.56	1.00	1869	3318

Table S2. Results of the hierarchical ordinal regression for beauty ratings using all trials. Readers are reminded that estimates shown are standardized as they represent beta estimates on the latent standardized normal scale. Intercepts denote partitions of the latent scale to derive cumulative probabilities [S2]. Est.Error = estimation error. CI = Credible Interval. ESS = effective sample size. ID = random intercept for each participant (n=34). Image_ID = random intercept for each stimulus (n=40). Observations = 2603.

Pleasure:

Predictors	Estimate	Est.Error	l-95% CI	u-95% CI	Rhat	Bulk_ESS	Tail_ESS
**Intercept[1]**	-2.37	0.14	-2.65	-2.09	1.00	1579	2752
**Intercept[2]**	-1.47	0.14	-1.74	-1.20	1.00	1510	2720
**Intercept[3]**	-0.74	0.14	-1.01	-0.48	1.00	1498	2844
**Intercept[4]**	-0.28	0.13	-0.54	-0.01	1.00	1491	2727
**Intercept[5]**	0.57	0.14	0.31	0.84	1.00	1502	2835
**Intercept[6]**	1.47	0.14	1.20	1.75	1.00	1585	3256
**Modality: Imagery**	-0.22	0.04	-0.31	-0.14	1.00	14675	7021
**Type: Face**	-0.82	0.12	-1.06	-0.58	1.00	2312	3569

Random effects							
**ID**	0.60	0.08	0.47	0.79	1.00	1936	3375
**Image_ID**	0.35	0.05	0.27	0.46	1.00	2989	3983

Table S3. Results of the hierarchical ordinal regression for pleasure ratings using all trials. Readers are reminded that estimates shown are standardized as they represent beta estimates on the latent standardized normal scale. Intercepts denote partitions of the latent scale to derive cumulative probabilities [S2]. Est.Error = estimation error. CI = Credible Interval. ESS = effective sample size. ID = random intercept for each participant (n=34). Image_ID = random intercept for each stimulus (n=40). Observations = 2588.

Correlation between vividness and similarity of evoked experiences across modalities

Predictors	Estimate	Est.Error	l-95% CI	u-95% CI	Rhat	Bulk_ESS	Tail_ESS
Intercept	-2.89	0.28	2.33	3.45	1.00	2774	4860
Vividness	-0.19	0.04	-0.28	-0.10	1.00	10211	7792
**Random effects**							
ID	0.97	0.17	0.67	1.35	1.00	1690	3294

Table S4.1. Correlation between the similarity of subjective experiences across stimulation modalities and vividness of the mental image. Unstandardized parameter estimates are shown. Est.Error = estimation error. CI = Credible Interval. ESS = effective sample size. ID = random intercept for each participant (n=34). Observations = 1130.

Parameter	Std. Median	95% CI
Intercept	2.00	[1.66, 2.38]
Vividness	-0.27	[-0.40, -0.14]

Table S4.2. Standardized parameter estimates for the correlation between the similarity of subjective experiences across stimulation modalities and vividness of the mental image. Parameters were standardized using the effectsize package [S3].

Hierarchical ordinal Bayesian analyses of aesthetic facets for highly vivid trials

Moving:

Predictors	Estimate	Est.Error	l-95% CI	u-95% CI	Rhat	Bulk_ESS	Tail_ESS
Intercept[1]	-2.47	0.16	-2.81	-2.16	1.00	1918	2940
Intercept[2]	-1.64	0.14	-1.92	-1.36	1.00	1847	2871
Intercept[3]	-0.99	0.14	-1.27	-0.72	1.00	1824	3067
Intercept[4]	-0.50	0.14	-0.78	-0.24	1.00	1791	2834
Intercept[5]	0.38	0.13	0.11	0.64	1.00	1786	2750
Intercept[6]	1.42	0.15	1.14	1.71	1.00	2100	2926

Modality: Imagery	-0.14	0.08	-0.29	0.01	1.00	6221	3558
Type: Face	-0.62	0.14	-0.89	-0.35	1.00	1858	2692
Random effects							
ID	0.48	0.09	0.33	0.68	1.00	1213	2123
Image_ID	0.33	0.07	0.21	0.47	1.00	1750	2425

Table S5. Results of the hierarchical ordinal regression for moving ratings using only highly vivid trials. Readers are reminded that estimates shown are standardized as they represent beta estimates on the latent standardized normal scale. Intercepts denote partitions of the latent scale to derive cumulative probabilities [S2]. Est.Error = estimation error. CI = Credible Interval. ESS = effective sample size. ID = random intercept for each participant (n=34). Image_ID = random intercept for each stimulus (n=40). Observations = 772.

<u>Beauty:</u>

**Predictors**	**Estimate**	**Est.Error**	**l-95% CI**	**u-95% CI**	**Rhat**	**Bulk_ESS**	**Tail_ESS**
Intercept[1]	-2.71	0.18	-3.07	-2.36	1.00	2928	3024
Intercept[2]	-1.80	0.15	-2.11	-1.51	1.00	2917	3430
Intercept[3]	-1.29	0.15	-1.59	-1.01	1.00	2769	3548
Intercept[4]	-0.89	0.14	-1.17	-0.62	1.00	2734	3485
Intercept[5]	0.08	0.14	-0.20	0.36	1.00	2719	3227
Intercept[6]	1.18	0.15	0.89	1.47	1.00	2801	3433
Modality: Imagery	-0.14	0.08	-0.29	0.01	1.00	7722	3348
Type: Face	-0.46	0.16	-0.80	-0.15	1.00	2281	2546
**Random effects**							
ID	0.40	0.08	0.27	0.58	1.00	1625	2593
Image_ID	0.43	0.07	0.30	0.58	1.00	1821	2930

Table S6. Results of the hierarchical ordinal regression for beauty ratings using only highly vivid trials. Readers are reminded that estimates shown are standardized as they represent beta estimates on the latent standardized normal scale. Intercepts denote partitions of the latent scale to derive cumulative probabilities [S2]. Est.Error = estimation error. CI = Credible Interval. ESS = effective sample size. ID = random intercept for each participant (n=34). Image_ID = random intercept for each stimulus (n=40). Observations = 783.

<u>Pleasure:</u>

**Predictors**	**Estimate**	**Est.Error**	**l-95% CI**	**u-95% CI**	**Rhat**	**Bulk_ESS**	**Tail_ESS**
Intercept[1]	-2.46	0.18	-2.80	-2.12	1.00	1535	2306
Intercept[2]	-1.78	0.16	-2.10	-1.46	1.00	1442	2174
Intercept[3]	-1.19	0.16	-1.51	-0.89	1.00	1367	2058
Intercept[4]	-0.74	0.16	-1.04	-0.43	1.00	1369	2162
Intercept[5]	0.20	0.15	-0.10	0.50	1.00	1392	2229
Intercept[6]	1.05	0.16	0.75	1.35	1.00	1481	2584
Modality: Imagery	-0.07	0.07	-0.22	0.07	1.00	5810	3712
Type: Face	-0.92	0.15	-1.21	-0.62	1.00	1857	2770
**Random effects**							
ID	0.56	0.10	0.40	0.78	1.00	1558	2416
Image_ID	0.39	0.07	0.27	0.54	1.00	1574	2266

Table S7. Results of the hierarchical ordinal regression for pleasure ratings using only highly vivid trials. Readers are reminded that estimates shown are standardized as they represent beta estimates on the latent standardized normal scale. Intercepts denote partitions of the latent scale to derive cumulative probabilities [S2]. Est.Error = estimation error. CI = Credible Interval. ESS = effective sample size. ID = random intercept for each participant (n=34). Image_ID = random intercept for each stimulus (n=40). Observations = 783.

Representational Similarity Analysis: partial model

Whole brain

Model	Eval ± SEM	p (against 0)	p (against NC)
Independent	0.464 ± 0.010	< 0.001	< 0.001
Modality	0.965 ± 0.010	< 0.001	0.484
Experience	0.044 ± 0.008	< 0.001	< 0.001
Type	0.071 ± 0.013	< 0.001	< 0.001
Exp|Type	0.081 ± 0.013	< 0.001	< 0.001
Mod|Exp|Type	0.624 ± 0.006	< 0.001	< 0.001
Mod|(Exp|Type)/2	0.835 ± 0.003	< 0.001	< 0.001
Mod|(Exp|Type)/3	0.908 ± 0.005	< 0.001	< 0.001
Exp|(Mod|Type)/2	0.459 ± 0.007	< 0.001	< 0.001
Exp|(Mod|Type)/3	0.352 ± 0.007	< 0.001	< 0.001

Reward network

Model	Eval ± SEM	p (against 0)	p (against NC)
Independent	0.610 ± 0.016	< 0.001	< 0.001
Modality	0.800 ± 0.025	< 0.001	0.015
Experience	0.144 ± 0.014	< 0.001	< 0.001
Type	0.161 ± 0.014	< 0.001	< 0.001
Exp|Type	0.216 ± 0.016	< 0.001	< 0.001
Mod|Exp|Type	0.638 ± 0.007	< 0.001	< 0.001
Mod|(Exp|Type)/2	0.778 ± 0.013	< 0.001	< 0.001
Mod|(Exp|Type)/3	0.816 ± 0.016	< 0.001	0.007
Exp|(Mod|Type)/2	0.510 ± 0.008	< 0.001	< 0.001
Exp|(Mod|Type)/3	0.420 ± 0.010	< 0.001	< 0.001

Caudate nucleus

Model	Eval ± SEM	p (against 0)	p (against NC)
Independent	0.686 ± 0.016	< 0.001	< 0.001
Modality	0.746 ± 0.028	< 0.001	< 0.001
Experience	0.208 ± 0.019	< 0.001	< 0.001
Type	0.215 ± 0.021	< 0.001	< 0.001
Exp|Type	0.299 ± 0.021	< 0.001	< 0.001
Mod|Exp|Type	0.675 ± 0.010	< 0.001	< 0.001
Mod|(Exp|Type)/2	0.782 ± 0.015	< 0.001	< 0.001
Mod|(Exp|Type)/3	0.802 ± 0.019	< 0.001	0.004
Exp|(Mod|Type)/2	0.562 ± 0.013	< 0.001	< 0.001
Exp|(Mod|Type)/3	0.478 ± 0.014	< 0.001	< 0.001

Nucleus accumbens

Model	Eval ± SEM	p (against 0)	p (against NC)
Independent	0.677 ± 0.016	< 0.001	< 0.001
Modality	0.745 ± 0.027	< 0.001	< 0.001
Experience	0.208 ± 0.022	< 0.001	< 0.001
Type	0.194 ± 0.020	< 0.001	< 0.001
Exp|Type	0.285 ± 0.022	< 0.001	< 0.001
Mod|Exp|Type	0.662 ± 0.009	< 0.001	< 0.001
Mod|(Exp|Type)/2	0.772 ± 0.013	< 0.001	< 0.001
Mod|(Exp|Type)/3	0.795 ± 0.017	< 0.001	0.002
Exp|(Mod|Type)/2	0.553 ± 0.014	< 0.001	< 0.001
Exp|(Mod|Type)/3	0.472 ± 0.017	< 0.001	< 0.001

Table S8. Candidate model performance across regions of interest using the whole dataset. p-values are based on uncorrected t-tests. NC = noise ceiling; SEM = standard error of the mean.

Representational Similarity Analysis: whole dataset

Whole brain

Model	Eval ± SEM	p (against 0)	p (against NC)
Independent	0.465 ± 0.010	< 0.001	< 0.001
Modality	0.965 ± 0.010	< 0.001	0.492
Experience	0.044 ± 0.008	< 0.001	< 0.001
Type	0.072 ± 0.013	< 0.001	< 0.001
Mod|Exp|Type	0.624 ± 0.006	< 0.001	< 0.001
Mod|(Exp|Type)/2	0.835 ± 0.003	< 0.001	< 0.001
Mod|(Exp|Type)/3	0.908 ± 0.004	< 0.001	< 0.001
Exp|(Mod|Type)/2	0.459 ± 0.007	< 0.001	< 0.001
Exp|(Mod|Type)/3	0.352 ± 0.007	< 0.001	< 0.001

Reward network

Model	Eval ± SEM	p (against 0)	p (against NC)
Independent	0.610 ± 0.015	< 0.001	< 0.001
Modality	0.801 ± 0.024	< 0.001	0.012
Experience	0.144 ± 0.014	< 0.001	< 0.001
Type	0.161 ± 0.013	< 0.001	< 0.001
Mod|Exp|Type	0.638 ± 0.007	< 0.001	< 0.001
Mod|(Exp|Type)/2	0.778 ± 0.012	< 0.001	< 0.001
Mod|(Exp|Type)/3	0.816 ± 0.016	< 0.001	0.005
Exp|(Mod|Type)/2	0.510 ± 0.008	< 0.001	< 0.001
Exp|(Mod|Type)/3	0.420 ± 0.010	< 0.001	< 0.001

Caudate nucleus

Model	Eval ± SEM	p (against 0)	p (against NC)
Independent	0.687 ± 0.016	< 0.001	< 0.001
Modality	0.745 ± 0.028	< 0.001	< 0.001
Experience	0.208 ± 0.019	< 0.001	< 0.001
Type	0.216 ± 0.021	< 0.001	< 0.001
Mod|Exp|Type	0.675 ± 0.010	< 0.001	< 0.001
Mod|(Exp|Type)/2	0.781 ± 0.015	< 0.001	< 0.001

Mod|(Exp|Type)/3	0.801 ± 0.019	< 0.001	0.004
Exp|(Mod|Type)/2	0.562 ± 0.013	< 0.001	< 0.001
Exp|(Mod|Type)/3	0.478 ± 0.014	< 0.001	< 0.001

Nucleus accumbens

Model	Eval ± SEM	p (against 0)	p (against NC)
Independent	0.677 ± 0.016	< 0.001	< 0.001
Modality	0.744 ± 0.028	< 0.001	< 0.001
Experience	0.208 ± 0.023	< 0.001	< 0.001
Type	0.194 ± 0.021	< 0.001	< 0.001
Mod|Exp|Type	0.662 ± 0.009	< 0.001	< 0.001
Mod|(Exp|Type)/2	0.772 ± 0.013	< 0.001	< 0.001
Mod|(Exp|Type)/3	0.794 ± 0.018	< 0.001	0.003
Exp|(Mod|Type)/2	0.554 ± 0.015	< 0.001	< 0.001
Exp|(Mod|Type)/3	0.472 ± 0.018	< 0.001	< 0.001

Table S9. Candidate model performance across regions of interest using the whole dataset. p-values are based on uncorrected t-tests. NC = noise ceiling; SEM = standard error of the mean

Representational Similarity Analysis: highly vivid trials

Whole brain

Model	Eval ± SEM	p (against 0)	p (against NC)
Independent	0.483 ± 0.012	< 0.001	< 0.001
Modality	0.972 ± 0.005	< 0.001	0.099
Experience	0.059 ± 0.009	< 0.001	< 0.001
Type	0.073 ± 0.013	< 0.001	< 0.001
Mod|Exp|Type	0.637 ± 0.007	< 0.001	< 0.001
Mod|(Exp|Type)/2	0.847 ± 0.003	< 0.001	< 0.001
Mod|(Exp|Type)/3	0.919 ± 0.002	< 0.001	< 0.001
Exp|(Mod|Type)/2	0.475 ± 0.008	< 0.001	< 0.001
Exp|(Mod|Type)/3	0.368 ± 0.009	< 0.001	< 0.001

Reward network

Model	Eval ± SEM	p (against 0)	p (against NC)
Independent	0.667 ± 0.017	< 0.001	< 0.001
Modality	0.723 ± 0.041	< 0.001	0.041
Experience	0.241 ± 0.042	< 0.001	< 0.001
Type	0.202 ± 0.032	< 0.001	< 0.001
Mod|Exp|Type	0.673 ± 0.021	< 0.001	< 0.001
Mod|(Exp|Type)/2	0.771 ± 0.025	< 0.001	0.076
Mod|(Exp|Type)/3	0.787 ± 0.029	< 0.001	0.296
Exp|(Mod|Type)/2	0.565 ± 0.025	< 0.001	< 0.001
Exp|(Mod|Type)/3	0.493 ± 0.030	< 0.001	< 0.001

Caudate nucleus

Model	Eval ± SEM	p (against 0)	p (against NC)
Independent	0.716 ± 0.022	< 0.001	0.002
Modality	0.626 ± 0.047	< 0.001	0.003
Experience	0.281 ± 0.050	< 0.001	< 0.001
Type	0.235 ± 0.047	< 0.001	< 0.001
Mod|Exp|Type	0.659 ± 0.024	< 0.001	< 0.001
Mod|(Exp|Type)/2	0.721 ± 0.028	< 0.001	0.014
Mod|(Exp|Type)/3	0.721 ± 0.034	< 0.001	0.031
Exp|(Mod|Type)/2	0.581 ± 0.030	< 0.001	< 0.001
Exp|(Mod|Type)/3	0.514 ± 0.035	< 0.001	< 0.001

Nucleus accumbens

Model	Eval ± SEM	p (against 0)	p (against NC)
Independent	0.698 ± 0.016	< 0.001	< 0.001
Modality	0.662 ± 0.048	< 0.001	0.010
Experience	0.252 ± 0.044	< 0.001	< 0.001
Type	0.218 ± 0.032	< 0.001	< 0.001
Mod|Exp|Type	0.653 ± 0.028	< 0.001	< 0.001
Mod|(Exp|Type)/2	0.732 ± 0.035	< 0.001	0.042

Mod|(Exp|Type)/3	0.740 ± 0.039	< 0.001	0.091
Exp|(Mod|Type)/2	0.565 ± 0.032	< 0.001	< 0.001
Exp|(Mod|Type)/3	0.493 ± 0.035	< 0.001	< 0.001

Table S10. Candidate model performance across regions of interest using only highly vivid trials. p-values are based on uncorrected t-tests. NC = noise ceiling; SEM = standard error of the mean

Model evaluation

Model name	ELPD difference	SE difference
Model_mod_type_rand	0.0	0.0
Model_type_rand_acat_cs(mod)	-2.3	3.8
Model_mod_type_rand_acat	-2.6	2.2
Model_rand_acat_cs(mod)_cs(type)	-6.3	3.9
Model_mod_type	-17.6	6.4
Model_mod	-43.9	10.4
Model_null	-44.0	10.6

Table S11. Assessment of model performance using leave-one-out cross-validation [S2]. The best-performing model is plotted first. Lower expected log predictive density (ELPD) [S4] compared to the winning model highlights worse performance overall. Model names denote which regressors were included. Null: none; Mod: Modality (perception vs imagery); Type: Type (face vs artwork); Rand; random intercept for each participant and each stimulus; acat_cs(): category-specific effect.

Data S1: Vividness of Visual Mental Imagery Questionnaire

Taken from the original publication by Marks in 1973 [S5].

For items 1-4, think of some relative or friend whom you frequently see (but who is not with you at present) and consider carefully the picture that comes before your mind’s eye.

Item
1. The exact contour of face, head, shoulders and body.
2. Characteristic poses of head, attitudes of body, etc.
3. The precise carriage, length of step, etc., in walking.
4. The different colours worn in some familiar clothes.

Visualize a rising sun. Consider carefully the picture that comes before your mind’s eye.

Item
5. The sun is rising above the horizon into a hazy sky.
6. The sky clears and surrounds the sun with blueness.
7. Clouds. A storm blows up, with flashes of lightning.
8. A rainbow appears.

Think of the front of a shop which you often go to. Consider the picture that comes before your mind’s eye.

Item
9. The overall appearance of the shop from the opposite side of the road.
10. A window display including colours, shapes and details of individual items for sale.
11. You are near the entrance. The colour, shape and details of the door.

12. You enter the shop and go to the counter. The counter assistant serves you.
Money changes hands.

Finally, think of a country scene which involves trees, mountains and a lake. Consider the picture that comes before your mind's eye.
Item
13. The contours of the landscape.
14. The colour and shape of the trees.
15. The colour and shape of the lake.
16. A strong wind blows on the trees and on the lake causing waves.

Data S2: Adapted and translated version of the Vividness of Visual Mental Imagery Questionnaire

Schließen Sie die Augen. Denken Sie an eine mit Ihnen verwandte oder befreundete Person, die Sie häufig sehen (aber die im Moment nicht bei Ihnen ist), und betrachten Sie sorgfältig das Bild, das Sie vor Ihrem geistigen Auge sehen. Bewerten Sie anschließend die folgenden Aspekte danach, wie anschaulich Sie diese vor Ihrem geistigen Auge gesehen haben:

Die genaue Kontur von Gesicht, Kopf, Schultern und Körper.
überhaupt nicht anschaulich 1 2 3 4 5 äußerst anschaulich

Charakteristische Kopfhaltung, Körperhaltungen, etc.
überhaupt nicht anschaulich 1 2 3 4 5 äußerst anschaulich

Die genaue Haltung, Schrittlänge etc. beim Gehen.
überhaupt nicht anschaulich 1 2 3 4 5 äußerst anschaulich

Die verschiedenen Farben der Kleidung, die die Person häufig trägt.
überhaupt nicht anschaulich 1 2 3 4 5 äußerst anschaulich

Denken Sie daran, dass Sie vor einem Laden stehen. Betrachten Sie sorgfältig das Bild, das Sie vor Ihrem geistigen Auge sehen. Bewerten Sie anschließend die folgenden Punkte.

Das Gesamtbild des Ladens von der gegenüberliegenden Straßenseite aus.
überhaupt nicht anschaulich 1 2 3 4 5 äußerst anschaulich

Eine Schaufensterdekoration mit Farben, Formen und Details von einzelnen Verkaufsartikeln.
überhaupt nicht anschaulich 1 2 3 4 5 äußerst anschaulich

Sie sind in der Nähe des Eingangs. Die Farbe, Form und Details der Tür.
überhaupt nicht anschaulich 1 2 3 4 5 äußerst anschaulich

Sie betreten den Shop und gehen zur Theke. Die Thekenkraft bedient Sie. Geld wechselt den Besitzer.
überhaupt nicht anschaulich 1 2 3 4 5 äußerst anschaulich

Data S3: Original version of the Aesthetic Experience Scale

Taken from the original publication by Silvia and Nusbaum [S6].

Please write down areas of the arts you encounter most often in your daily life:

How often do you . . . (1: never or rarely - 7: nearly always)

_______ feel absorbed and immersed
_______ completely lose track of time
_______ feel chills down your spine
_______ get goose bumps
_______ feel like you’re somewhere else
_______ feel like your hair is standing on end
_______ feel like crying
_______ feel touched
_______ feel detached from your surroundings
_______ feel a sense of awe and wonder

Data S4: Adapted and translated version of the Aesthetic Experience Scale

Bitte wählen Sie die Kunstdomäne aus, die bei Ihnen die stärksten Emotionen bzw. das stärkste ästhetische Empfinden auslöst:

- Musik hören
- Videos schauen (Fernsehen, Kino, Youtube, etc)
- Bildende Kunst (Gemälde, Malerei, Kunstfotografie, etc.)
- Literatur
- Tanzaufführungen
- Theater
- Eigenes kreatives Schaffen
- Andere

Wie häufig sind Sie der von Ihnen angegebenen Kunstdomäne in etwa ausgesetzt?

- täglich
- mehrmals die Woche
- einmal die Woche
- ein- bis zweimal im Monat
- alle paar Monate
- ein- bis zweimal im Jahr

noch seltener

Bitte beantworten Sie die folgenden Fragen in Bezug auf die oben von Ihnen ausgewählte Kunstdomäne. Dabei sollen Sie beurteilen, wie oft die folgenden emotionalen und physischen Zustände bei Ihnen ausgelöst werden, wenn Sie dieser Kunstdomäne ausgesetzt sind: (1: niemals oder kaum, 7: fast immer)

Labels (1-7):
1: *niemals oder kaum*, 7: *fast immer*

Wie oft fühlen Sie sich vollkommen eingenommen von und versunken in ihr Erleben?
niemals oder kaum 1 2 3 4 5 6 7 fast immer

Wie oft verlieren Sie vollkommen das Zeitgefühl?
niemals oder kaum 1 2 3 4 5 6 7 fast immer

Wie oft fühlen Sie einen Schauer über Ihren Rücken laufen?
niemals oder kaum 1 2 3 4 5 6 7 fast immer

Wie oft bekommen Sie eine Gänsehaut?
niemals oder kaum 1 2 3 4 5 6 7 fast immer

Wie oft haben Sie das Gefühl ganz woanders zu sein?
niemals oder kaum 1 2 3 4 5 6 7 fast immer

Wie oft haben Sie das Gefühl, dass Ihnen die Haare zu Berge stehen?
niemals oder kaum 1 2 3 4 5 6 7 fast immer

Wie oft haben Sie das Gefühl, weinen zu müssen?
niemals oder kaum 1 2 3 4 5 6 7 fast immer

Wie oft fühlen Sie sich tief berührt?
niemals oder kaum 1 2 3 4 5 6 7 fast immer

Wie oft fühlen Sie sich von Ihrer Umgebung losgelöst?
niemals oder kaum 1 2 3 4 5 6 7 fast immer

Wie oft fühlen Sie sich von Ehrfurcht und Staunen ergriffen?
niemals oder kaum 1 2 3 4 5 6 7 fast immer

Data S5: Comprehension Questions

When should you not move?

Explain the scale from 1-7 of the question “How beautiful was the image?”.

What is aesthetically moving?

What is pleasure?

What do you need to do when you hear the tone?

What does the question ”How vivid was your imagery?” assess?

Supplemental references

S1. Tierney, N., and Cook, D. (2020). Expanding tidy data principles to facilitate missing data exploration, visualization and assessment of imputations. Preprint at arXiv.

S2. Bürkner, P.-C., and Vuorre, M. (2019). Ordinal Regression Models in Psychology: A Tutorial. Advances in Methods and Practices in Psychological Science *2*, 77–101. https://doi.org/10.1177/2515245918823199.

S3. Ben-Shachar, M.S., Lüdecke, D., and Makowski, D. (2020). effectsize: Estimation of Effect Size Indices and Standardized Parameters. Journal of Open Source Software *5*, 2815. https://doi.org/10.21105/joss.02815.

S4. Vehtari, A., Gelman, A., and Gabry, J. (2017). Practical Bayesian model evaluation using leave-one-out cross-validation and WAIC. Stat Comput *27*, 1413–1432. https://doi.org/10.1007/s11222-016-9696-4.

S5. Marks, D.F. (1973). Visual Imagery Differences in the Recall of Pictures. British Journal of Psychology *64*, 17–24. https://doi.org/10.1111/j.2044-8295.1973.tb01322.x.

S6. Silvia, P.J., and Nusbaum, E.C. (2011). On personality and piloerection: Individual differences in aesthetic chills and other unusual aesthetic experiences. Psychology of Aesthetics, Creativity, and the Arts *5*, 208–214. https://doi.org/10.1037/a0021914.
